# “Will it be enough to be respected?”: understanding personal, institutional, and societal harms and benefits of genomics research

**DOI:** 10.1007/s12687-026-00941-4

**Published:** 2026-09-10

**Authors:** Emerson Dusic, Devin Madden, Isabella E. Rios, Arjee Restar, Tiq Milan, Jodie Patterson, David R. Pletta, Sima Rabinowitz, Michelle A. Ramos, Rodrigo A. Aguayo-Romero, Kellan Baker, Carol R. Horowitz, Sari L. Reisner

**Affiliations:** 1 Department of Epidemiology, University of Michigan, University of Michigan School of Public Health, 1415 Washington Heights, Ann Arbor, 48109 MI USA; 2 Center for Social Epidemiology and Population Health, University of Michigan, Ann Arbor, MI USA; 3 Institute for Health Equity Research, Icahn School of Medicine at Mount Sinai, New York, NY 10029 USA; 4 Department of Population Health Science and Policy, Icahn School of Medicine at Mount Sinai, New York, NY USA; 5 Department of Behavioral and Social Sciences, Yale University, New Haven, CT USA; 6 The Human Rights Campaign, New York, NY USA; 7 The Institute for Health Research and Policy at Whitman Walker, Washington, DC USA

**Keywords:** Transgender, Genomics, Public health, Stigma, Identity, Genetics

## Abstract

**Background:**

Transgender Identity Genomics Research (TIGR)—research examining potential associations between genetic factors and transgender, nonbinary, and gender diverse (trans) identities—takes place in a complex landscape with significant potential to either benefit or harm trans communities. As public and political scrutiny of trans communities intensifies, the stakes and potential impacts of TIGR are heightened and ethical, legal and social implications must be considered. To better understand how those who would be most affected by TIGR view such research, we explored trans adults’ perceptions of TIGR in the current environment and how they perceive TIGR may impact their futures.

**Methods:**

Using a community-based participatory research approach, we partnered with a trans-led Executive Stakeholder Board to conduct in-depth interviews with 31 trans adults in the United States between June-December 2024 and conducted a Reflexive Thematic Analysis.

**Results:**

Participants (mean age=34 years, range=19-73 years; 61% people of color; 39% nonbinary, 39% transgender women, 22% transgender men) identified possible benefits of TIGR such as personal affirmation, increased social acceptance, and improved access to gender-affirming healthcare. They also expressed concerns about potential harms, including further stigmatization, discrimination, and pathologization of trans identities. While most viewed research in trans communities (including TIGR) as a net positive, some participants felt it less useful compared to addressing more urgent, material challenges trans communities face.

**Conclusion:**

Our findings underscore that TIGR, like all scientific research, is shaped by the sociopolitical environment. It is imperative that TIGR researchers engage meaningfully and ethically with trans communities to minimize potential harms and center community needs.

**Supplementary Information:**

The online version contains supplementary material available at https://doi.org/10.1007/s12687-026-00941-4.

## Introduction

Transgender Identity Genomics Research (TIGR) encompasses genomics and genetic studies that examine potential relationships between genetic factors and aspects of gender identity, sex development, and gender diversity (Rajkovic et al. 2022; Theisen et al. 2024a, see Table 1 for glossary of terms used in this paper). TIGR has complex ethical, legal, and social implications given its potential impact on transgender, nonbinary, and gender diverse (trans) communities whose health and lived experiences have historically been pathologized and marginalized by medical and scientific institutions (Lowik 2018; Vincent 2018). Proponents of TIGR emphasize its potential to advance understanding of human diversity, expand clinical care for trans individuals, and promote greater inclusion of trans people in genomics research (Cura et al. 2025; Polderman et al. 2018; Rajkovic et al. 2022; Theisen et al. 2024a, b). Critics caution that TIGR risks further pathologizing trans identities, reinforcing binary or deterministic views of gender, and enabling misuse of findings to justify discrimination or medical gatekeeping (Levin et al. 2023; Rajkovic et al. 2022).

**Table 1:** Glossary of relevant terms

Cisgender: Describes people whose gender is the same as their sex assigned at birth
Gender: A social construct describing an individual’s internal sense of being a man, woman, both, or neither of these. Often based on culture-bound conventions, roles, and behaviors. Someone’s gender identity may or may not align with their sex assigned at birth, whether or not they are transgender
Gender-affirming healthcare (GAHC): Healthcare that affirms one’s gender identity, including but not limited to hormone replacement therapy and gender-affirming surgery
Gender diverse: An umbrella term often used in academic literature to describe populations with a broad range of gender expression
Gender dysphoria: A medical diagnosis included in the Diagnostic and Statistical Manual of Mental Disorders: DSM-5, replacing “gender identity disorder” in an effort to de-pathologize trans identities. Also commonly used colloquially and in academic literature to describe experiences of distress among trans and gender diverse populations resulting from discord between one’s gender identity and their physical body and/or others’ perceptions of their gender. Not all trans people experience gender dysphoria
LGBTQ: Lesbian, gay, bisexual, transgender, and queer; an umbrella term used to represent communities of sexual and gender minorities
Nonbinary: An umbrella term used to describe people who identify as neither a man nor woman, both a man and a woman, or something outside of these binary constructs altogether. Nonbinary people may or may not identify as transgender
Queer: An umbrella term used to represent communities of sexual and gender minorities. Historically used as a slur, the term has been largely reclaimed as a positive, and often political, identity
Sex: A scientific and social construct used to categorize individuals based on biological characteristics such as hormonal milieu, reproductive organs, external genitalia, secondary sex characteristics, and/or chromosomal composition. This is often ascribed at birth as either male, female, or intersex
Sexual orientation: A person’s identity in relation to what genders they are sexually and/or romantically attracted to. Importantly, this is a distinct concept from gender identity
Transgender (trans): Describes people whose gender differs from their sex assigned at birth
Transphobia: The expression of prejudice, hostility, and/or violence towards trans people

Although karyotyping studies of trans individuals to look for differences in sex chromosomes date back to the 1960 s (Hoenig and Torr 1964), the first family or twin study was not published until the 1990 s (Buhrich et al. 1991). This research has suggested some degree of heritability for gender diversity, although findings have been inconsistent (Buhrich et al. 1991; Conabere et al. 2025, 2025; Lippa and Hershberger 1999; Polderman et al. 2018). In the 2000 s, research expanded to include candidate gene studies (Bentz et al. 2008; Levin et al. 2023), which have largely evaluated hormone receptors genes but have had limited replicability (Ashley and Harley 2023; Bentz et al. 2008; Ghojazadeh et al. 2025; Levin et al. 2023; Polderman et al. 2018). Notably, definitions and measures of gender and gender identity have shifted over time and vary across cultural contexts; consequently, earlier studies relied on clinical diagnoses and categories that do not align with contemporary understandings of gender identity.

These early TIGR efforts emerged in the United States (U.S.) during a period of limited public understanding and visibility of trans individuals; widespread and legal discrimination of trans individuals in housing, employment, and healthcare on the basis of gender identity; and low engagement with trans communities in research (Tracey 2022). In the 2010 s, growing public advocacy, legislative and technological innovations, and broader visibility coincided with major advancements in genetics. TIGR subsequently evolved to include whole exome sequencing studies that suggested potential associations between hormone receptor genes and gender identity development (Liu et al. 2025; Theisen et al. 2019). Yet, these studies faced methodological constraints, including small sample sizes, failure to be consistently replicated, and limited generalizability. Additionally, none of these studies have described efforts to engage with trans communities.

Alongside growing visibility and advocacy came an increase in anti-trans legislation, intensified efforts to limit trans rights, and an increase in discriminatory practices against trans individuals, particularly since 2024 (Trans Legislation Tracker 2026). While increased public attention on trans issues may foster broader awareness and understanding of trans individuals and trans identities, it may also heighten the potential for TIGR to be misinterpreted or weaponized in ways that exacerbate stigma and harm. In this context, meaningful engagement with trans communities is crucial to ensuring TIGR proceeds in ways that are socially accountable, ethically responsible, and that establish trans peoples’ health as a priority.

Few published studies have examined trans individuals’ perspectives on TIGR. Two qualitative interview studies (Hammack-Aviran et al. 2022; Rajkovic et al. 2022) and two quantitative surveys (Theisen et al. 2019; Thomas et al. 2020) among lesbian, gay, bisexual, transgender, and queer (LGBTQ) individuals found that while participants expressed concern about potential harms, such as reinforcing essentialist narratives or enabling eugenic misuse, they also recognized TIGR’s potential benefits to support medical care and affirm trans identities. All studies called for additional, community-engaged research to better understand trans perspectives on TIGR’s potential benefits and risks, its social and ethical implications, and the broader context within which research is conducted. Therefore, we sought to examine how trans adults in the U.S. perceive TIGR in the current sociopolitical and scientific landscape, and how they believe it may impact their futures.

## Methods

### Participants and Procedures

Data came from the Transforming Genomics Study, which explored perspectives on TIGR and developed guidelines to inform future genomics research with trans populations, employing a community-based participatory research approach (Horowitz et al. 2009). All work was conducted in partnership with the study’s Executive Stakeholder Board (Board), a group of geographically and professionally diverse trans community members and advocates, clinicians, health equity scholars, and genomics researchers.

A seven-member study team—with a majority LGBTQ scientists, alongside allied researchers—conducted in-depth interviews with 31 trans adults between June and September 2024. Eligible individuals were over age 18 years of age, lived in the U.S., and identified as transgender, nonbinary, or gender diverse. The study team, in collaboration with the Board, developed the interview guide, which was piloted with two participants who were included in analysis. Topics included TIGR, acceptability of genetic research, and policy context (Supplement 1, Interview Guide) and were used to stimulate discussion regarding stigmatization, medicalization, biological and genetic essentialism, and gender identity.

Board members distributed recruitment materials among professional, healthcare, and community-based organizations, and personal networks, but did not recruit more broadly online to prevent bots and fraudulent responses. Flyers instructed interested individuals to contact the study email. Our research team then sent potential participants the link to a short screening survey on Mount Sinai’s HIPAA-compliant REDCap system to determine eligibility. We purposively sampled participants to ensure maximum variation (Coyne 1997) in the study population with regards to race, gender identity, sex assigned at birth, and geography. Recruitment took place until we reached theoretical saturation (Hennink and Kaiser 2022).

Two authors (ED, IR) conducted the interviews over Zoom, each lasting approximately 60 min. Although it was not explicitly included in the interview guide, interviewers openly discussed their identities and positionality with participants as this subject arose (i.e., some interviewees asked the interviewer if they were trans so that participants could change their language from ‘my community’ to ‘our community’). At the end of each interview, we asked participants to complete a brief demographic survey online. Interviewers took detailed notes during interviews and, following interviews, participated in reflexive journaling on potential themes and general impressions. A third-party service professionally transcribed the recordings. We ensured anonymity by removing demographics from participant quotes in this paper, based on a suggestion from a participant. Two academic IRBs approved the study and determined it was exempt (Mount Sinai #23–01264 and the University of Michigan #HUM00252081).

### Data Analysis

Three authors (ED, IR, DM) coded interviews in Atlas.ti (version 25.0.1) using a deductively defined codebook based on the interview guide and reflexive journaling. We used Reflexive Thematic Analysis (RTA), with a rigorous reconciliation process to ensure reliability and to identify and organize key themes (Braun and Clarke 2022, 2024). The flexibility of RTA allowed analysts to take an inductive approach, despite defining codes deductively, in which participants’ thoughts and analysts’ latent interpretations guided the development of themes. Following analysis, we invited participants to a results share-back session where the lead authors engaged in reflexive participant collaboration (Motulsky 2021). The authors presented the research findings and engaged in open dialogue with participants to ascertain their interpretation of the findings and how their perspectives may have changed since the time of the interviews. This conversation was not recorded and is not included in the dataset but has informed the discussion section of this manuscript. We summarized data from the brief demographic survey to contextualize qualitative findings.

## Results

### Participant demographics

Table 2 provides a summary of participant demographics with additional details in Supplement 2. Among the 31 participants, average age was 34 years (range = 19–73), 39% were nonbinary, 39% transgender women, 22% transgender men and 61% were people of color. Among the 14 interviewees who self-reported a disability (45%), attention deficit (50%), autism (43%), health-related disability (13%), and a mental health condition (13%) were the most reported conditions. Participants had high education levels, 20 (65%) completed bachelor’s or graduate degrees. Our recruitment targeted all 50 states; the 31 enrolled participants represented a continuum of trans-related policy environments, ranked from discriminatory to protective (defined by Movement Advancement Project) (Movement Advancement Project, n.d.).

**Table 2:** Demographic characteristics of participants (*n* = 31)

Characteristics	M ± SD or *n* (%)
**Age**	33.6 ± 12.2
**Gender**	
Nonbinary	12 (38.7%)
Transgender man	7 (22.6%)
Transgender woman	12 (38.7%)
**Two-Spirit**^+^	
No	30 (96.8%)
Yes	1 (3.2%)
**Sex assigned at birth**	
Female	18 (58.1%)
Male	13 (41.9%)
**Intersex**	
No	24 (77.4%)
Yes	1 (3.2%)
Don’t know	6 (19.4%)
Race/Ethnicity^*^	
American Indian or Alaskan Native	1 (3.2%)
Asian	5 (16.1%)
Black or African American	7 (22.6%)
Hispanic or Latino/a/x/e	4 (12.9%)
Middle Eastern or North African	2 (6.5%)
Native Hawaiian or Pacific Islander	1 (3.2%
White	16 (51.6%)
**Education**	
Some high school or less	1 (3.2%)
Completed high school (or GED)	2 (6.5%)
Some college or associate degree	8 (25.8%)
Complete college/bachelor’s degree	4 (12.9%)
Technical/vocational school	0 (0.0%)
Some graduate school	4 (12.9%)
Complete graduate school	12 (38.7%)
**Income**	
≤$24,999	8 (25.8%)
$25,000-$49,999	5 (16.1%)
$50,000-$74,999	7 (22.6%)
$75,000-$99,999	4 (12.9%)
$100,000-$124,999	3 (9.7%)
$125,000-$149,999	1 (3.2%)
≥$150,000	2 (6.5%)
Don’t know	1 (3.2%)
**Geographical Location**	
Midwest	4 (12.9%)
Northeast	11 (35.5%)
South	9 (29.0%)
West	6 (19.4%)
Choose not to answer	1 (3.2%)
**Gender Identity Policy Climate**	
High	17 (54.8%)
Medium	3 (9.7%)
Fair	2 (6.5%)
Low	0 (0.0%)
Negative	8 (2.5%)
Choose not to answer	1 (3.2%)
**Disability Status**	
No	15 (48.4%)
Yes	14 (45.2%)
Choose not to answer	2 (6.5%)

^*^Individuals could select more than one response

^+^Participants were only shown question if they indicated they were American Indian or Alaskan Native. Denominator for percentage is total study population

### Thematic overview

We generated five interconnected themes through RTA, reflecting a diversity of opinions on how TIGR may impact trans communities at personal, institutional, and societal levels: (1) Personal Validation, Self-Discovery, and the Limits of a Potential Genetic Explanation; (2) Access to Care, Gatekeeping, and Medicalization; (3) Public Understanding, Proof, and Pathologization; (4) Recognition, Risk, and the Politics of Representation; and (5) Perceived Relevance and Competing Priorities.

### Personal Validation, self-discovery, and the limits of a potential genetic explanation

Several participants expressed the belief that a genetic marker for trans identities would be personally validating and could help alleviate the shame or grief they experienced early in their transition while coming to understand their gender identity. Knowing there was a genetic factor that contributed to their being trans would offer peace of mind and affirm the sense that they were “*born this way*” (Table 3, Q1.1) or that their identity was predetermined (Table 3, Q1.2). For these participants, this was the biggest benefit and the “*North Star*” of TIGR (Table 3, Q1.3). Other participants said that it would not personally matter to them, while acknowledging that identification of genetic factors associated with trans identity could be personally meaningful for others, especially early in their gender transition (Table 3, Q1.4).

**Table 3:** Exemplary quotes organized by theme

Theme	Quote
Personal Validation, Self-Discovery, and the Limits of a potential Genetic Explanation	Q1.1: “I think if you sat people down and said that you were born this way, that it’s just a process of coming into your identity… I know kids now who are two and three years old who—because their parents are informed—are being allowed to live their identity at a very, very young age. Right? And if you said, well, the reason you know this is because your body is programmed to know this and how we’re programmed to know this is because of our gene sequencing, chromosomes, then people would be, like, ‘Oh, absolutely.’” -P12 Q1.2: “I think the time where it would have been the most helpful to me personally to know would have been very early in transition. To have some sense of… ‘Yeah, this is really happening, this is really real.’ You know? I can maybe imagine a world where like… I don’t know, this still feels too close to dystopia, but here’s a way that it would help: I started crossdressing before I acknowledged that I was trans. And at the time I thought it was a fetish and I thought it was bad and I had all kinds of shame about it. If I could have, say, gotten tested [for a genetic marker for trans identity] then and realized, ‘Oh, no, I’m doing this because I’m trans,’ that might have saved me some years and some grief and some regret.” -P24 Q1.3: “I need to just kind of reemphasize. I think there are a lot of trans folks for whom it could provide a certain peace of mind. And so insofar as keeping a positive outcome in the back of mind for this research, I think that’s maybe a little bit of a North Star. You know, [researchers] are providing this benefit and if not, maybe let’s press pause because you know there’s a lot of risks. So let’s make sure we’re providing benefits.” -P24 Q1.4: “I think that a lot of trans people would be vulnerable to wanting to know, wanting to have an external authority validate whether they were genuinely trans and whether they would regret transitioning or not at a certain point. So I feel like, yeah, a lot of people would be very tempted to get whatever type of answer they could about that prior to actually transitioning medically or socially in order to have a verified way of seeing whether they would regret it or not and seeing whether they could possibly be happy without doing it or would like have to do it or else they would die or something.” -P13 Q1.5: “I think it’s really cool that I figured this out about myself. I did all the work to make me comfortable in my own body, and that took a lot of introspection. If it was something that I just inherited, […] I feel like it almost takes the magic away.” -P16 Q1.6: “INTERVIEWER: Let’s say they did all of this research, and the conclusion of the research was ‘We didn’t find any genetic markers, there’s no genetic explanation.’ What do you think is the impact of that on the public perception of trans people? PARTICIPANT: Well, the radical right would probably use it against us and say, ‘See! It is a lifestyle.’ Blah, blah, blah, blah, blah. But I mean, I’ve been in therapy for decades. And for two years prior to my transition, my therapist was asking me to start looking through the lens of gender dysphoria. […] It’s definitely in your brain, in your heart, in your being. And fighting it, hiding it, blocking it, keeping it in the closet and tormenting it, it’s just not worth it. If there’s no genetic reason for it, no genetic marker that’s clearly indicated—and it would have to be a study that can be proven over and over again that this is actually a fact—then some people are going to use it for bad. And some people are going to say, ‘Well, I don’t care what it says.’ And I think I’d be more on the—of I don’t care what it says.” -P11 Q1.7: “INTERVIEWER: Do you believe that there might be, at least in some small part, a genetic explanation for gender identity? PARTICIPANT: Yes is the answer. I have read--there are a lot of very suggestive scientific studies out there. Everything from psychology, meaning psychological testing that’s done and that are designed, and everything from young children up to adults, that suggest that trans people, their brains are wired in a certain way.” -P17 Q1.8: “I think each person interprets the culmination of all of the pieces of [biology] in line with their social existence and sort of comes to an understanding. I don’t know if it’s one particular piece [of evidence] over another. I’ve also seen things tying it to hormones and don’t know how legitimate that is, like neonatal hormones. I just don’t have the know-how to really judge this information, but there’s enough questions there that leads me to think that it’s probably not an—‘Oh, this part of your brain determines gender identity’—but rather, X, Y, Z other thing is influencing me and making me… What the hell makes someone gender fluid? I don’t think parts of my brain are shrinking and growing, I don’t know. But it’s also a reality that I’m experiencing, and I don’t think it’s one that’s entirely based in social reality without something in folks’ bodies as a validation.” -P3 Q1.9: “Just keeping in mind that someone’s own self-conception of their gender and the sorts of choices that they want to make about how to exist in their gendered way in society in terms of like their name, how they dress themselves, what, if any, medical transition things they do, et cetera, are extremely personal choices that I feel like have to be sort of enshrined as like a fundamental bodily autonomy issue that people have the right to make those decisions about themselves. They are the experts on themselves, and they have more insight than anyone else can about themselves and it’s not a situation where an outside authority needs to assess what the correct decision for someone is. It’s just the person themselves need to decide. […] And I think that any research that would infringe on that conception of gender and transness as a very self-defined thing based on what someone wants for their life and wants for their existence is harmful until proven otherwise and it would need to meet a very high threshold of why it would be useful before it would make sense to have resources allocated to any type of research about who was trans.” -P13 Q1.10: “I don’t think that just because many people have the experience of this seeming like an immutable thing about themselves that they’ve known their whole lives means that there is a genetic component to it that it would be possible to isolate or manipulate or assess in somebody. I think some people are gay and some people are trans and some people are neither and it doesn’t need to be this big thing of like, ‘Well, why would people be that way?’ People have always been like this, generally. We haven’t always used certain language to refer to people. People haven’t always known things about themselves. But these types of diversity of human experience have always been the case.” -P13
Access to Care, Gatekeeping, and Medicalization	Q2.1: “INTERVIEWER: Then one of the benefits that you said you could think of was for trans youth–if they know that they have this gene or genetic marker, it may help them access something like puberty blockers faster. Right? PARTICIPANT: Yeah, which obviously would be great. But I just feel that the harm outweighs the benefit. For some people who know [that they are trans at a young age], wonderful, get them into the care pipeline. But even if we had that testing, […] if you needed to submit a genetic test before getting that care, I just think that would go very, very wrong.” -P15 Q2.2: “I feel like with people wanting to find a genetic basis for gayness, it wasn’t with the same sort of undercurrent that I feel like there is with transness of figuring out who ‘deserves’ to be trans. Because transness has always had this situation of medical gatekeepers deciding whether someone deserves to be trans or not and gayness does not have that. No one is like do you deserve to have gay sex or not? Because you don’t have to do that. And even with something like gay marriage, no one is like, ‘Well, are you a real gay person who really deserves to get in a gay marital relationship or should you not be allowed to do this?’ So I just feel like… because of this thing with transness where there’s like this external authority that determined whether you’re “really’ trans or not or should be allowed to transition, it’s like especially harmful with the transness compared to gayness to be trying to find the genetic basis of it.” -P13 Q2.3: “I’m 23 years old and I can’t even access trans healthcare because I live with my parents. Transmedicalism is not fun for me because I can’t access care. You know, hearing stuff like [you need to access GAHC to be really trans] from your own community, it’s like, well, are you going to pay for my surgeries? Are you going to pay for my bills? No? Okay, then shut the fuck up.” -P18 Q2.4: “As soon as we say there is a biological marker for being trans then what happens if someone is having those feelings and they don’t test for that biological marker, either because they don’t have it or because the test is erroneous for some reason? Are they suddenly now not valid as a trans person? Do they not get to transition? Are they somehow lesser than any folks who do have a certain biological marker? Do we say that you’re only allowed to play with your gender if you have that biological marker and everyone else has to conform? I think there are answers out there that are useful to us then I think I wouldn’t even want those answers to be used in the service of denying freedom and liberation and [exploration].” -P24
Public Understanding, Proof, and Pathologization	Q3.1: “If someone is trans and has that [genetic] marker, that would serve as a validation of identity. Especially for religious groups that really don’t accept trans people, I could see that being a gateway to acceptance, that it’s something biological that they were born with, which could be from God, whatever religious stuff you would like to insert there. So, it does have the capacity to help with acceptance as well.” -P15 Q3.2: “Overall, hate is hate, and it’s not going to be kind of a magic fix. And it could lead to, like I said earlier, it could lead to the idea of, ‘Oh, so it’s something that we can get rid of. It’s still unnatural. It’s a mutation,’ kind of thing, instead of it just being a gene like another gene that makes your eyes blue. But I think it could kind of go either way.” -P27 Q3.3: “Will it be enough to be respected, if [TIGR research does] find anything? And if they don’t find anything, does that mean you’re just going to call [trans people] mental?” -P6 Q3.4: “I feel like it’s. this is the new way of this–I call it psychology or like medicalization of being trans. Cis[gender] people or non-trans community will see us as less than, as people who cannot control themselves because there’s already a stigma about us. Now you’re saying that ‘Okay, these people that you have a stigma [against] are biologically this way,’ and it reminds me of I don’t know, this study of like race and genes, like the way that they “explained” how some races are inferior. I feel like, because there exists a stigma. And they already think that trans people are less than. There is this cissexism going on in society. I feel like naturalizing this transness makes some trans people medically less than, which is very dangerous.” -P1 Q3.5: “PARTICIPANT: I do think probably some, especially transphobic, people think that at least some trans people falsely believe they’re trans because they have no ability to know themselves because of autism specifically. And I could see them–like autism is genetically predetermined–so I guess I could see that. Like if people are like, ‘Oh, transness is just this freaky thing that people with developmental disabilities come up with.’ It would not be that the transness itself was genetically based necessarily but that the transness was because of -- INTERVIEWER: Like some kind of disorder or something. PARTICIPANT: Or like, yeah, maybe some other mental health condition.” -P13 Q3.6: “I don’t really want to talk to cis people about [a “trans gene”] usually for the same reason why, when there was much more of a public focus about like a quote-unquote gay gene, I also like didn’t really want to hear the opinions of straight people on that because I don’t know, it very quickly starts going down a like, and then we can prevent it-kind of road. Even people who profess not to be homophobic or transphobic will say, ‘I’m not saying that you should prevent people from being trans or gay, because that’s bad. But discrimination is hard, and maybe some parents aren’t equipped to raise LGBTQ kids. So like, maybe it’s a good thing if we can prevent people from being trans or gay.’ I hate that. It’s just like straightforward eugenics. So I try not to talk about this kind of thing with cis people because often it kind of turns into me doing an impromptu trans 101 education thing, which I’m not always interested in doing. I do a lot of that in my professional life and my personal life, but sometimes I just don’t want to.” -P21 Q3.7: “There are so many people that think being queer is fake or a phase. Having a genetic component to it does kind of give it more validity medically as being a condition that can help spur further rights and research.” -P4 Q3.8: “It goes back to the clarity. Once you get a better understanding of the root of something, I think that it opens up a door. Could be negative and positive. But I believe it could be something positive, because if I’m not mistaken, I want to say back in the 70 s or the 80 s, they looked at [trans-ness] as some type of mental illness, if I’m not mistaken. And so, when you can get down to that, to the bottom of that, that can maybe enlighten them and say, ‘Hey, well, this is not some type of mental illness, this is genetic.’ And put a little more respect on the transgender.” -P8 Q3.9: “It just bothers me a lot. That I have to show proof to people that I am truly trans, or I am okay to be trans. Just leave. Let me be.” -P1
Recognition, Risk, and the Politics of Representation	Q4.1: “If there is a trans gene and I test positive for the trans gene, cool. But it wouldn’t add anything to my life. It wouldn’t change anything about the way I live my life. The only [positive] result [is that it] would validate me to people who wouldn’t have respected me unless they had this reason, you know? And those people’s respect is not something I’m interested in. ” -P31 Q4.2: “No one really talks about those type of things in the [trans] community. It’s just out of the [trans] community, because [cisgender people] need some type of proof. They need proof of why it’s happening to you and not them. So this genetic study probably is a very good thing, because at worst case it’ll get a lot of people to just shut up and finally prove there is something genetically connected.” -P7 Q4.3: “Historically, sometimes alignment with either nature or pathology has been a positive temporary way of approaching justice work. Always temporary, but frequently positive. So maybe there are people out there who are like, if we nail down this sort of natural biological conclusion of ‘Your baby was always going to be trans because here’s the marker that said that in their genes and you have to accept them because it’s your genes that did it.’ That could be positive and historical like parallels suggest that that is not the be all end all of justice.” -P3 Q4.4: “So if you don’t have this genetic marker, is it going to be like, ‘Well, you’re not really trans?’ Are we going to get a whole new level of, I don’t know if I’m saying it right, but the people who accuse people of being trans for clout or whatever […] I could see that happening and it getting divisive. ‘Go pull up your genetics and let’s see if you’re really trans’ or whatever.” -P28 Q4.5: “If [conservatives] have their essentialist arguments that everything is biological, then [if research were to find that] transness is not biological, then they can argue, or something like that. And the problem with politics is that is not very academically driven. I can definitely see arguments coming through that they are saying that ‘Okay, trans people are not even biologically, something valid.’” -P1 Q4.6: “I can see it being used against the group of people that it’s trying to help. History tends to repeat itself, and there’s already history of similar things happening. So, while I do think [TIGR] is really important and can help the individual a lot, I can also see it being used for that larger policy conversation. Right now, I think there’s just not enough [research] for them to be able to make a policy or bring [TIGR] in to that conversation. I think if it was further along, that might be kind of a different story though.” -P10 Q4.7: “I think a lot of the bathroom bills and a lot of the anti-trans bills that are going around are part of that, part of eugenics, part of we do not want this population in public life or around. So we’re going to try and legislate you out of existence. And we’ve seen that before with other groups of people. Black people, Indigenous people, anyone who the people in power in the U.S. was like, we don’t want you here. We don’t want to see you or we want your labor, but we don’t want to have you as part of your society. So I really do feel like a lot of the anti-trans bills are also eugenicist in nature.” -P28 Q4.8: “I feel like forcing it to be related to genetics feels like more threatening right now because of the way the world is. I mean, the fact that Texas is pulling lists of like people who have changed their gender, why? What exactly are you planning on doing with that? It can’t be good.” -P25 Q4.9: “I’m Jewish. So I’ll just go straight to that historical context, which is if there’s a marker for it and you can test for it then you can be selected at any stage of life. In my opinion we’re in a fight of democracy versus fascism right now, and if fascism wins and then we have a genetic marker for things. You know? Submit for your blood tests and then off you go. Right? So I think that there’s a possibility for dangerous situations when something like a blood test can be an indicator. It’s also dangerous when the test is ‘Because I say so.’ Right? You’re too young to remember. But you probably know the history of–there were ordinances about how many articles of clothing you could have that did not align to your assigned sex at work. If you violated that, then they’d arrest you. That was one of the ways that they rounded people up at bars and speakeasys and carted them off to jail overnight was because a woman had on a necktie and a vest and those are both male.” -P12 Q4.10: “Depending on where you fall on your political views around trans people, [TIGR] will sway you. If you’re one of those more indifferent, could care less about trans people and you think in very simple way and you don’t think critically, there are some data that show you’ll be like, ‘Cool. Okay, I guess this is legitimate, like trans can’t help themselves. This is not made up. They’re not crazy. There’s a biological reason.’ So there might be more acceptance during this political climate or there could be people who believe that trans people are mentally ill, so they will say, ‘See? This is why they’re not normal, because there is a genetic reason. The gene was supposed to be expressed like this normally, but it didn’t happen, and that’s why they’re weird or that’s why they’re that way. I think that same data could be interpreted and used differently, even if it’s the same number or whatnot, by the people with a different agenda or beliefs.” -P5
Perceived Relevance and Competing Priorities	Q5.1: “INTERVIEWER: Thinking about all those different research priorities you just talked about, where does finding a ‘trans gene’ or genetics of transness fall in this like priority list? Is it lower, higher priority? Where do you see that? PARTICIPANT: Honestly, I see that in a lower priority. Because there are things that are much more important than finding out why in the hell somebody is trans. Like, you shouldn’t even care. If you’re not trans, you shouldn’t be worrying about it. […] so it’s low. I think that that would be nice and it would be an extra incentive for our community, but it’s very low on the totem pole. We need better housing, better healthcare, better food in the world. […] There’s a lot of stuff that’s much, much more important than that. However, that would be very nice to prove our side of the argument. Because right now it kind of seems like we may be losing the argument. But that’s -- it’s very unimportant, but we still want to win. We just want people to see us as normal people.” -P9 Q5.2: “I would rather people be able to have like research being done towards possibly gender-affirming care—something like that—rather than them trying to figure out what makes us trans because it’s like a chicken before the egg. I’m already trans. I’d rather you figure out how to help me.” -P18 Q5.3: “Working at [research institution] I remember saying to my boss and my coworkers, ‘The problem is that we can’t just make [trans people] feel like numbers. We can’t just make them feel like pincushions.’ You know what I’m saying? They have lots of things going on in their personal lives before they think about doing [genetic] tests and all, before they’re thinking about coming in and receiving any kind of services. Right now, they’re trying to find housing, probably trying to find food, probably trying to find healthcare. And these are the things that we need to be helping them address first while knowing that the end goal is still to get them tested. And I believe that helping them set these goals and achieve them will make it easier to get to your end result.” -P14 Q5.4: “Yes, we need to reallocate resources to effectively help our community, and also there are always different fields that need to progress. This field just requires more money. Now we could even argue that disproportionately, if we’re pouring more money into this and less into services, then I would think there’s definitely some weight to what community people are saying. But let’s say if—I’m just making this up—the same amount of funding goes into social services for trans folks and the other half goes to research, I would be fine with that. There will always be people like, ‘Well, no, 100% of [funding] should going into services because people need that.’ Yes. At the same time, I feel like it’s not okay for us to think black or white. Because that’s very much of a survival mentality of if we don’t do this, everyone’s going to die, kind of a thing, rather than we can still do both, knowing that we’re in it for a long haul.” -P5 Q5.5: “INTERVIEWER: Have you heard about genetics and biology being discussed in policy conversations? PARTICIPANT: Broadly. I think people definitely try to use research and policy as a general kind of thing. The more we understand, the easier it is to, on the one hand, weaponize, on the other hand, support [trans individuals], so to speak. I haven’t heard a lot of specific policies recently, but just in general, the idea of using research in order to change or to create policy that could be both for and against trans people, especially trans youth, is a big one that I have heard more of. I haven’t heard a lot of kind of biology being used.” -P27 Q5.6:“I think the goal is to get information rather than action. I think that’s also more important. Like in the same thing of like this gene is associated with trans people rather than this gene *causes*, ‘cause I think this gene causes can cause stuff like, ‘Hey, what if we remove that gene? Hey, what if we affect that gene? But hey, if you get this, it means you 100% have to have transness.’ So I think caution and I guess being careful about wording and how they want to interpret stuff is also a big deal.” -P19 Q5.7: “There has to be co-leadership. [Scientists] also should be working with a communication team, where they have to be very strategic about, ‘what is it that what is the main message we want to send?’ It’s not just that we want to find out if trans people, it’s not their fault that they’re trans. That shouldn’t be the primary messaging […] what we’re not saying is that the data will legitimize us as humans. Regardless of the data, we still need human rights, all the basic needs that we’re struggling for. That piece is where I think the co-director, co-leadership, and vision has to be coming from the [trans] community. Then they can’t see it as like either/or, but both and, that these two [messages] are complementary.” -P5

Still others feared that TIGR could also undermine the personal and transformative process of self-discovery, with one individual describing the “*magic*” of finally being able to put language to their internal feelings (Table 3, Q1.5). Understanding their gender identity involved introspection, emotional work, and personal exploration—all of which they considered integral to their sense of self. As a result, a genetic test indicating they were *not* trans, or the possibility that TIGR might find no genetic correlates for gender identity, would not disrupt their life trajectories or understanding of themselves (Table 3, Q1.6). They expressed a deep sense of self-knowledge and confidence in their identities, emphasizing that they did not question whether their transness was valid or measurable, nor need any additional extraneous proof.

Several participants believed that an underlying biological basis for trans identity is likely. One participant recollected reading studies that suggested a neurological basis for trans identity, explaining, *“trans people*,* their brains are wired a certain way”* (Table 3, Q1.7). Some participants noted that there are trans individuals who know they are trans from an early age, even if they initially lack the language to describe it, which they viewed as evidence of a natural or predetermined aspect of gender identity. Others disagreed and said that looking to biology to answer questions about trans identity was oversimplistic. These participants viewed essentialist explanations of gender as restrictive, arguing that they impose fixed categories of who is and is not trans and constrain individuals’ ability to self-identity or evolve in their gender identity and expression over time. They emphasized that being trans is not only a matter of biology, but also of lived experience, reflection, and self-determination (Table 3, Q1.8). One participant noted that decisions about social and medical transition remained deeply personal and could not be reduced to genetics or biology (Table 3, Q1.9). The same participant also discussed how diversity of human experience and gender expression has been present in all societies throughout time and questioned the need to seek biological explanations for these differences (Table 3, Q1.10).

Most participants recognized that diversity of perspectives and lived experiences within trans communities can influence one’s perspective on this topic. Everyone agreed that all people should have the autonomy to explore and understand their gender identity in their own way. As several stated, trans people are “*experts on themselves*” (Table 3, Q1.9) and should have the autonomy to self-identify without needing medical or genetic validation, in the same way cisgender people don’t need to prove their identities. Participants described exploration as a fundamental aspect of the human experience, emphasizing that all people undergo personal processes of self-discovery.

### Access to care, gatekeeping, and medicalization

Participants expressed mixed views about whether finding genetic correlates of trans identity would decrease or increase access to gender-affirming healthcare (GAHC, referring to healthcare that affirms one’s gender identity, including but not limited to hormone replacement therapy and gender-affirming surgery). Some believed that if a genetic test for trans identity existed, insurance providers might be more willing to cover the cost of GAHC for individuals who tested positive. Others feared that such testing could prompt insurers to classify trans identity as a pre-existing condition, thereby introducing new financial or logistical barriers (Table 3, Q2.1).

Several participants drew parallels to the historical search for a potential genetic marker for sexual identity, which shaped their views on TIGR’s potential harms and benefits. However, one participant highlighted an important distinction between a hypothetical trans gene versus gay gene, noting that trans identities are often medicalized and defined in relation to the medical care a person receives or is deemed to “*deserve*” (Table 3, Q2.2). This participant argues that the pursuit of potential genetic correlates for trans identity, therefore, carries more weight in respect to medical gatekeeping and further restricting access to healthcare under the guise of medical gatekeeping.

Other participants similarly viewed TIGR as promoting a medicalized conception of trans identity, wherein the identification of a biological marker would imply that GAHC is the necessary and expected treatment. They described this perspective as reductive and naïve, warning that it risks undermining trans peoples’ rights to self-determination and medical autonomy (Table 3, Q2.3). For example, one individual raised concern that a negative genetic test result for someone who self-identifies as trans would thwart their access to GAHC (Table 3, Q2.4).

### Public understanding, proof, and pathologization

When asked about the potential benefits of TIGR, some participants believed that identifying a genetic marker for transness could prove that trans identities are real, potentially fostering greater public acceptance. They felt this could be particularly meaningful in religious contexts or in countries where trans people face violence and discrimination based on gender expression, offering what one participant described as a potential “*gateway to acceptance*” (Table 3, Q3.1). Many participants viewed more research in trans communities (including TIGR) as a net positive, expressing hope that greater scientific understanding of trans identities would promote broader public understanding and acceptance.

On the other hand, participants expressed deep concern about the broader public discourse and expression of the view that being trans is not real or that trans people are mentally ill. Many participants referenced how transness and queerness more broadly have historically been classified as a disease, leading to harmful attempts at cures or corrections. While some hoped TIGR could help challenge these misconceptions, others cautioned that such research would not be a “*magic fix*” for the discrimination and stigma trans people face in daily life (Table 3, Q3.2). Many feared that if TIGR failed to identify genetic components associated with trans identity, transphobic individuals (defined as those expressing prejudice or hostility toward trans people) might misuse the findings to claim that trans identities are fabricated or that trans people are making it up. One participant captured this tension by asking, *“Will it be enough to be respected*,* if [TIGR research does] find anything? And if they don’t find anything*,* does that mean you’re just going to call [trans people] mental?”* (Table 3, Q3.3). While this participant questioned whether identifying a genetic basis for gender identity would provide sufficient ‘proof’ to increase respect for trans people, they also worried that null findings could be interpreted in ways that reinforce pathologizing beliefs.

Others worried that, conversely, if TIGR were to identify a genetic correlate, trans identity could be framed as a mutation, furthering discrimination and even raising fears of institutionalization (Table 3, Q3.4). Similarly, a few participants expressed apprehension that any genetic correlates of gender identity could be linked to genetic correlates of mental illness, further stigmatizing trans identities. They worried that gender incongruence has historically been pathologized and associated with mental illness in ways that have disproportionately harmed trans communities, people of color, and people with disabilities (Table 3, Q3.5).

Participants voiced significant concern that the search for a potential genetic basis for transgender identity perpetuates a long history of scientists pathologizing LGBTQ communities, and nearly all participants expressed worry that TIGR could eventually be used to justify eugenic practices targeting trans people, such as selective abortion, genetic modification of embryos, or social pressure discouraging trans reproduction. These fears were grounded in the historical pathologization of trans communities, who have long been exploited and discriminated against by medical systems, as well as real-world examples like selective abortion of fetuses with disabilities or intersex traits. One participant described the emotional toll of attempting to discuss the potential of a genetic basis for trans identity with cisgender people in their life noting how candidly people began to express opinions that sounded as if they favored eugenic practices, even when these people did not consider themselves to be transphobic (Table 3, Q3.6).

### Recognition, risk, and the politics of representation

Several participants felt that TIGR would have little impact on their daily lives and that the existence of a potential genetic basis for gender identity didn’t fully matter (Table 3, Q4.1). Some also expressed the view that TIGR primarily serves to appease cisgender, heterosexual perspectives of trans individuals. As one participant explained, they rarely discuss the idea of a potential genetic basis for trans identity with other trans people, but cisgender individuals often ask why they think they are trans, questions they find burdensome and intrusive. Although many did not care whether a supposed trans gene exists, they acknowledged that TIGR could still have value if it were to help convince cisgender people that gender identity is biologically or genetically based (Table 3, Q4.2). Many also said that even if genetic factors did contribute to gender identity, gender identity is likely multifactorial and influenced by other factors such as environmental interactions and gene expression.

Participants overwhelmingly agreed that TIGR cannot be separated from the current political climate, particularly amidst the escalating anti-trans rhetoric and policy. Some believed that demonstrating a genetic or biological basis for being trans could have a positive effect on society by framing transness as an inherent, natural variation rather than a personal choice, thereby decreasing hostility, violence, and discrimination against trans communities. Many participants found this idea of genetic predetermination compelling, especially for audiences such as religious groups that may otherwise reject trans identities. Alternately, one described this idea as precarious, and a “*temporary way of approaching justice work*” (Table 3, Q4.3) that could ultimately create divisions within trans communities over who is considered “*really trans*” (Table 3, Q4.4) A few participants noted that politics are “*not very academically driven*” (Table 3, Q4.5) so even if genetic correlates of trans identity were identified, it would not change the beliefs or actions of anti-trans policymakers, who they viewed as driven by ideology rather than science.

Many expressed concern that, at present, the potential harms of TIGR may outweigh its potential benefits. As one participant explained, even well-intended research can be used to harm marginalized communities (Table 3, Q4.6). There was some consensus that efforts to identify a genetic basis for trans identity could inadvertently reinforce harmful thinking and ideas about trans people. Some participants feared that if a genetic marker for trans identity were discovered, it could be misused to identify, monitor, or target trans individuals, particularly within an increasingly hostile political climate. Several cited existing systems of surveillance, such as government tracking of gender marker changes on IDs and hospital data breaches, as evidence that being identifiable as trans already carries risk. They also drew historical parallels, noting that trans individuals have long faced punishment, imprisonment, and state-sanctioned violence when their identities were exposed.

Many participants connected TIGR to a broader historical pattern of using pseudoscience and eugenics to justify harm against marginalized groups, including enslavement, institutionalization, sterilization, and genocide of racialized populations and people with disabilities. Several expressed fear that trans people could similarly be targeted for genocide or sent to concentration camps, viewing current conservative and anti-trans policies as moving in that direction. One referenced the persecution and murder of trans people during the Holocaust as historical precedent. Others warned that contemporary anti-trans rhetoric and legislation increasingly reflect eugenic ideologies and could escalate into mass violence (Table 3, Q4.7).

### Perceived relevance and competing priorities

Several did not perceive clear clinical utility in TIGR. For example, one participant who had previously undergone BRCA testing noted that their provider recommended the test to guide future care decisions, whereas knowing whether someone is trans through a genetic test would not similarly inform clinical management of follow-up care. However, participants acknowledged that TIGR could have value if it contributed to precision medicine approaches that improve GAHC for trans individuals (for example, understanding how exogenous hormones interact with their individual genetic profiles). They also supported TIGR if it led to research efforts that address health-related outcomes and advanced the wellbeing of trans communities, such as generating increased support for trans advocacy organizations and trans equality.

Even among participants who recognized the potential benefits of TIGR, most believed that funding could be better directed toward addressing more immediate needs within trans communities (Table 3, Q5.1 and Q5.2) such as housing, healthcare access, and mental health support. One participant remarked that meeting these basic needs could increase trans people’s ability to participate in TIGR, explaining that many would be focused on securing such day-to-day essentials before considering genetic testing (Table 3, Q5.3). Others expressed hope that if TIGR were to identify a genetic component of gender identity, it might lead to increased funding in social services and related research benefitting trans communities. One participant emphasized that supporting trans people should not be viewed as a choice between funding TIGR or direct services—both are important (Table 3, Q5.4). Participants expressed the view that balancing investment in both research and community support offers the most sustainable path toward advancing trans health and wellbeing.

Despite concerns about resource allocation and potential harms stemming from the research, most participants did not call for stopping TIGR. Instead, they emphasized the need to support the research with strong ethical frameworks, particularly in how the research is structured, conducted, and communicated. One participant said, *“The more we understand*,* the easier it is to*,* on the one hand*,* weaponize and*,* on the other hand*,* support [trans individuals]*” (Table 3, Q5.5). Another stressed the importance of presenting findings as nuanced associations rather than causal claims (Table 3, Q5.6). Across interviews, participants agreed that trans individuals should be meaningfully involved in TIGR at every stage, from study inception and data collection to interpretation and dissemination (Table 3, Q5.7).

## Discussion

**Fig. 1:**
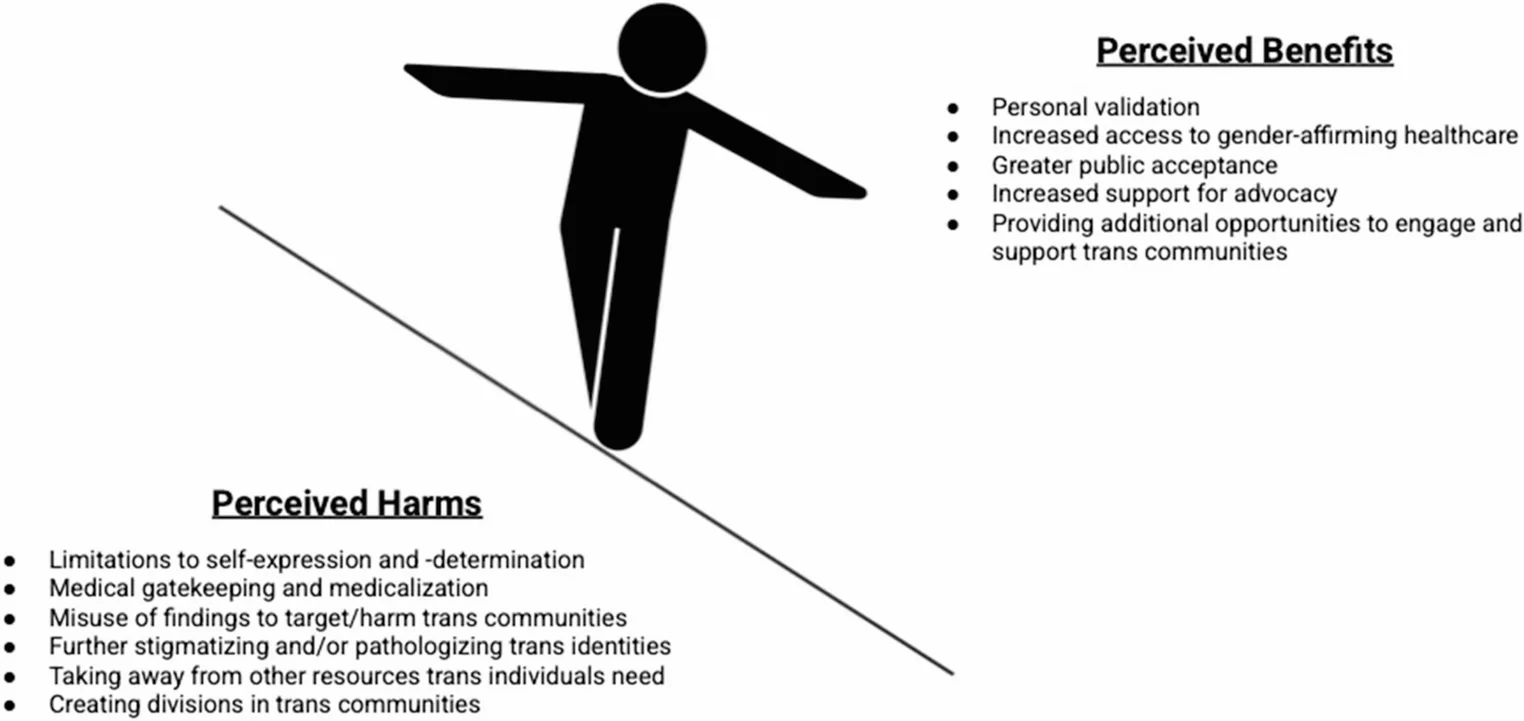
Summary of participants’ perceived harms and benefits of transgender identity genomic research

Our study explored how trans people perceive TIGR in the current sociopolitical and scientific environment, and how they envision its potential future impacts. Participants’ perspectives reflect their understanding of TIGR as delicate balance between producing meaningful benefits and posing serious risk of harms (Fig. 1). They expressed the view that the potential impacts of this research are heightened in the current sociopolitical and scientific environment. Perceived benefits of TIGR included personal validation, expanded access to GAHC, improved public understanding of trans identities, additional value in conducting more research with trans communities, and objective proof of transness to support trans rights. Potential risks included further pathologizing or stigmatizing trans identities, reinforcing narratives of transness as a mental illness, promoting narrow definitions of what it means to be trans, limiting access to GAHC, increasing medical gatekeeping, potential for eugenic practices, and fueling political violence such as anti-trans policies.

Our finding that participants believe TIGR could enable discrimination, even as it enabled self-validation and public acceptance, were corroborated in prior qualitative and quantitative studies (Hammack-Aviran et al. 2022; Rajkovic et al. 2022; Theisen et al. 2024a; Thomas et al. 2020). Participants in Rajkovic et al. (2022) and Hammack-Aviran et al. (2022) similarly expressed fears that TIGR could enable discrimination even as they recognized its potential for self-validation and broader public acceptance. Prior studies also found differing perspectives on whether TIGR might increase or restrict access to GAHC. The persistence of these themes over time and in shifting political environments suggests that such tensions are enduring and fundamental to how trans individuals engage with TIGR. Overall, these findings point to the importance of employing meaningful, thoughtful, and community-engaged approaches to ensure that TIGR’s potential benefits are realized while minimizing harms.

Participants’ fear of TIGR potentially harming trans communities was grounded in a history of trans communities being harmed by medical and research institutions, as well as examples of prior eugenic practices targeting trans, disabled, and intersex communities. There is a well-documented history of trans individuals being targeted by eugenic strategies, including sterilization, imprisonment, and institutionalization (Lowik 2018; Ordover 2023; Stern 2024). For example, eugenic field workers would identify individuals whose gender expression deviated from expected norms to recommend for sterilization (Cartwright 2021). The trajectory of this research, from 20th century eugenics to contemporary genetics studies, parallels the treatment of other socially stigmatized traits. For example, individuals were identified by eugenic fieldworkers based on their measured intelligence were labeled “feeble-minded,” and, more recently, genetic research seeking potential correlates of intelligence and educational attainment have directly resulted in acts of violence towards marginalized communities (Molteni 2022). Additionally, trans individuals have been subjected to institutional and interpersonal violence when accessing GAHC and other forms of medical care, including discrimination from medical providers and policies that limit ability to access GAHC (Hanssmann 2023; James et al. 2016). The concerns of participants in our study suggest that importance of TIGR researchers to explicitly address historical injustices, anticipate the ethical and clinical ramifications of their work, and engage trans communities in developing safeguards from future harm.

Additionally, participants expressed concern that TIGR findings could be stripped of nuance and appropriated to support political or ideological agendas. This concern is particularly salient given the current surge in anti-trans legislation—from 153 bills introduced in 2021, to 701 in 2024 (at the time the interviews took place), 1,022 in 2025, and nearly 800 in the first 6 months of 2026 (Trans Legislation Tracker 2026)—and U.S. Presidential Executive Orders that target so-called “gender ideology” and intentionally (mis)use genetic and biological terminology (Defending Women from Gender Ideology Extremism and Restoring Biological Truth to the Federal Government 2025; Redfield and Chokshi 2025). Genetic researchers studying gender identity must carefully consider the political and social implications of their work. TIGR investigators should anticipate how their findings may be interpreted or misused by the public and policymakers. Consistent with previous studies (Hammack-Aviran et al. 2022; Rajkovic et al. 2022; Theisen et al. 2024a; Thomas et al. 2020), our findings underscore the complexity of TIGR and reinforce the scientists’ responsibility to communicate results thoughtfully. As Jamal et al. (2024) explain, integrating nuance and expansive context-informed understandings of sex and gender into genomics research not only reduces the risk of misappropriation, but also produces more inclusive and accurate science that better reflects human diversity. Conversely, oversimplified and decontextualized interpretations can be used to legitimize discriminatory policies. For example, several states have redefined sex as binary, objective, and immutable to exclude trans individuals from nondiscrimination protections and restrict access to public accommodations (Movement Advancement Project, n.d.). In line with Jamal et al. and our participants’ perspectives, we reiterate the importance of intentional, community-engaged, and context-sensitive approaches to TIGR that advance scientific understanding while supporting trans communities.

Participants emphasized that trans identity could not be reduced to genetics or biology alone. They noted that variations in gender expression have appeared throughout history and across cultures (Heyam 2022) suggesting that societal, cultural, and historical factors play a significant role in how gender identity emerges and is understood. Similarly, both research results and their societal impact are likely to differ substantially across cultures due to differences in how gender identity is understood and the level of support trans communities receive. Further, the definitions and measurements of different aspects of gender identity—and specifically gender dysphoria, which is often the outcome variable in TIGR—have evolved over time, ideally moving away from pathologizing individuals toward centering the impact of sociopolitical environments and medical systems (Davy and Toze 2018; Sumerau 2023). In early editions of the *Diagnostic and Statistical Manual of Mental Disorders* (DSM), gender and sexual diversity were categorized as sexual deviancy (Cartwright 2021). Although homosexuality was removed from the DSM in 1973, diagnoses describing gender identity disorder were introduced in the 1980 s, reflecting efforts to identify psychological and/or biological causes for trans identity (Drager 2023). Gender identity disorder was later replaced by gender dysphoria with the publication of the DSM-5 in 2013 (Davy and Toze 2018), a change intended, in part, to reduce stigma by describing experiences of distress among trans populations resulting from discord between one’s gender identity and their physical body and/or others’ perceptions of their gender. However, the continued inclusion of gender dysphoria in the DSM remains contested; while some argue that it reinforces pathologization of trans people, others emphasize its importance for facilitating access to GAHC.

This discussion raises a broader question: if trans identities were not pathologized and stigmatized within society, would TIGR exist in its current form? While genetic research could potentially contribute to our understanding of gender identity, it is critical to recognize that any potential genetic or biological basis is unlikely to be straightforward or isolated from sociopolitical and cultural context. For example, while participants often categorized the identification of a potential genetic marker for trans identity as a binary yes/no with complete penetrance, it is very unlikely that this would be the case; in fact, results from TIGR utilizing whole exome sequencing have identified several potential genetic markers (Theisen et al. 2019). Therefore, TIGR must account for and engage with the profound influence of culture, evolving definitions of gender identity, the evolving complexity of biology and genetics, and the powerful role of society in shaping both scientific inquiry and lived experience.

Lastly, while many participants acknowledge that TIGR could offer personal validation for themselves or others, many others said their support for TIGR stemmed primarily from the hope that it might silence skeptics. Participants recognized the potential benefits of TIGR and viewed these as secondary to more urgent needs within trans communities such as addressing the pervasive violence, discrimination, and material insecurity trans people face in their daily lives. Participants welcomed TIGR as a possible tool in this broader context but believed that it should not come at the expense of more immediate forms of support and protection. Importantly, participants did not view these pressing, material needs as reasons to halt TIGR altogether, but suggested that working with trans individuals to make sure their needs are met could also improve engagement with TIGR. Moving forward, researchers should consider for whom and for what purpose their studies are intended and ensure genuine collaboration with trans communities that centers their priorities (Minalga et al. 2022; Veale et al. 2022).

The findings we have highlighted reflect the perspectives and positionalities of the study team, which consisted of a majority of trans researchers whose experiences and views informed the development of the research questions and the interpretation of results. Although trans individuals are often grouped into a singular, homogenous community in research, it is important to recognize the diversity of perspectives, experiences, and priorities across distinct trans communities. Accordingly, the findings from our study should not be interpreted as representative of the trans community as a whole. For example, our sample did not include trans youth, who are particularly vulnerable to political and structural violence and may hold different views on TIGR. In addition, our sample was largely well educated, and most participants resided in states with positive policy environments. Lastly, although we did not ask individuals in our sample how long they have openly identified as trans, our findings that TIGR may be particularly influential for individuals early in their gender transition warrants further investigation. Future research is needed to better understand the opinions and experiences of trans individuals who remain underrepresented or especially vulnerable, including trans youth and those living in states that have been significantly impacted by anti-trans policies. Future research should also investigate difference in perspectives on TIGR based on specific research methods utilized (i.e. twin studies versus whole exome sequencing). Additionally, given the underrepresentation of trans individuals in genomics research specifically and in health research more broadly, future research should examine how trans communities perceive the potential harms and benefits of allowing other areas of genetics research to advance while limited attention is granted to trans topics. Another limitation of our study is that data collection occurred prior to the 2024 U.S. election; given the escalation of anti-trans policies since this time, perspectives may have shifted. Strengths include a purposively diverse, nationwide sample of trans adults (over 50% of whom were people of color), enhancing the depth and complexity of perspectives. With few prior studies examining perspectives of TIGR among trans people (Hammack-Aviran et al. 2022; Jamal et al. 2024; Theisen et al. 2024a), this study contributes novel insights into how trans people perceive TIGR within its broader sociopolitical and scientific context helping to both shape and safeguard the emerging field.

As TIGR continues, future research should prioritize exploring how findings from TIGR intersect with lived experiences, policy, and clinical care and develop strategies to prevent misuse of research to further stigmatize and discriminate against trans communities. As participants noted, scientific research does not occur in a vacuum, but instead is shaped by, and has consequences for society, policy, and trans peoples’ lives. Our findings underscore the imperative for researchers to thoughtfully engage with the complexity of TIGR, collaborate directly with trans communities, prioritize the reduction of harm, and approach TIGR with caution and ethical responsibility. Ultimately, our findings reinforce the need for genomic research on trans identities to be pursued with rigorous ethical safeguards that prioritize autonomy, self-determination, and protection from harm.

## Supplementary Information

Below is the link to the electronic supplementary material.

Supplementary Material 1 (DOCX 38.5 KB)

Supplementary Material 2 (DOCX 19.2 KB)

## Data Availability

Data will be made available upon reasonable request. Access requires submission of a concept sheet for investigator review and completion of the necessary data use agreements between institutions.
