# Supplementary material for: “Will it be enough to be respected?”: understanding personal, institutional, and societal harms and benefits of genomics research
Source: J Community Genet. 2026 Sep 10;17(5):105. doi: 10.1007/s12687-026-00941-4 (PMC13562480; doi:10.1007/s12687-026-00941-4)
Supplement: Supplementary file 1 [file 12687_2026_941_MOESM1_ESM.docx]

**Trans/Forming Genomics Interview Guide**

**Overall Research Question:** How do transgender, nonbinary, and gender diverse (trans) individuals perceive genomic research related to gender identity?

**After Consent/and Layout of interview:**

Thank you for meeting with me/us today! Our team is interested in learning about how trans and gender diverse people feel research that looks at genes and the environment (genetic research) to try to better understand gender identity.

To make sure we’re on the same page before beginning the interview, I want to explain some words we’ll be using.

- We use the term trans to describe individuals who are transgender, nonbinary, and gender diverse. We know that this may not be the language you use in your personal life, and that not all nonbinary or gender diverse people identify as trans. We are only using the term trans so we go through the interview a little faster and don’t take up more of your time than we have to.
- When we say sex, we mean the physical traits that are usually linked to being male, female, or intersex.
- When we say gender identity, we mean one’s internal sense of being a man, woman, neither of these, both, or another gender(s).
- When we say genetic research, we mean the study of a person's genes and how their genes interact with each other and with other factors, like where we live and our behaviors, to impact our health.

Do you have any questions before we get started?

[Start recording]

**Introductory Questions/Gender Identity (5 min)**

- Can you tell me a little about yourself and why you were interested in this study?
- How do you describe your gender?

**Trans Identity and Genetic (30 min)**

- Do you believe there may be – at least in part – a genetic explanation for gender identity? Why or why not?
  - From your perspective, what is the purpose of this type of research?
  - Have you thought about or talked with anyone about this?
    - Who did you talk to about it? Is it easier to have these conversations with certain people?
      - Probe: friends, trans community members, family, doctors
    - What was it like to talk about it?
  - What, if anything, do you think are the risks (personal, medical, social, policy) of genetics research that focuses on trans identity or includes trans people?^a^
    - How did you arrive at that perspective?
    - Would that be helpful or harmful to trans people?
  - What, if anything, do you think are the benefits (personal, medical, social, policy) of genetics research that focuses on trans identity or includes trans people?^a^
    - How did you arrive at that perspective?
    - Would that be helpful or harmful to trans people?
  - If there is a genetic explanation for transgender identity:
    - Would you want to know? Why or why not?
    - How do would it impact how you perceive your own gender identity, if at all?
    - How would this impact the public perception on trans people, if at all?
  - What if there was no genetic explanation for gender identity? Would that be helpful or harmful to trans people- or impact public perception?
  - Do you think there is a sense of agreement among the trans community on whether there is a genetic explanation for transgender identity? Why or why not?
    - If there is a genetic explanation for transgender identity, do you think the trans community at large would want to know? Why or why not? If you’re unsure, why are you unsure?
  - How does your racial/ethnic identity affect your perspective on this? How and why? What about any other aspects of your identity?
- Do you believe this type of research should occur? Why or why not?
  - How else could this type of research impact trans people?
  - What concerns, if any, do you have about this type of research?
  - What advice would you give these researchers? What would you want them to know in order to maximize benefit and minimize harm?
- We’ve talked a lot about genetics and gender identity. Other researchers may be looking at other biological or physiological explanations for gender identity (hormones, neurological differences, etc.).
  - Do you believe there may be – at least in part – a biological or physiological explanation for gender identity? Why or why not?

**Acceptability of Genetic Research (10 min)**

For this next section of the interview, we are curious about your opinion on participation in genetic research more broadly.

- How can researchers best support trans people who take part in genetic research?
  - How might researchers or clinicians work with trans communities to increase trust around this type of work?
- Who should be doing or leading this type of research? Who should be included/participating in this type of research?
  - How do you see trans people being involved in this type of research?
  - How important for you is it for trans people to be involved in this research? Why?
  - Would you want to be a part of a team looking into this? Why or why not?
  - Would you want to be a participant in this type of research? Why or why not?
- What barriers do you think there are for the inclusion of trans people in genetic research?
- We are developing guidelines for people doing genetic research with trans people or who receive the results of this type of research. What would you want them to know?
  - Probes: collection of sex/gender, collection of transgender identity, how sex and gender are used in research studies
- What kind of genetic research, if any, do you think is needed for trans community?

**Policy (10 min)**

- In your view, how are trans people responding to national conversations about transgender rights?
  - Probes: Gender affirming healthcare, bathroom bans, **legal sex definitions**, **gender marker changes**
  - How have you heard genetics and biology being discussed in these larger policy conversations?
    - IF NO: Have you heard of policies that have been proposed to redefine sex in order to exclude transgender people from policy protections?
    - How do you feel about policies such as sports or bathroom bans that focus on anatomical descriptions of transgender people?
    - How, if at all, has the policy environment affected how you think about genetic research?

**Debrief/Wrap Up (5 min)**

- What do you want researchers and policymakers to know about how work on genetics and gender identity could impact trans people?
- Is there anything we didn’t get to cover about gender identity and genetic research that you think is important to discuss?
- Do you have any questions for me?
- Would it be okay to contact you in the future if we have questions about your responses?

Thank you for participating! While I am preparing your gift card, would you be able to complete our demographic survey?

^a^Question order switched every other participant
