# Supplementary material for: “Will it be enough to be respected?”: understanding personal, institutional, and societal harms and benefits of genomics research
Source: J Community Genet. 2026 Sep 10;17(5):105. doi: 10.1007/s12687-026-00941-4 (PMC13562480; doi:10.1007/s12687-026-00941-4)
Supplement: Supplementary file 2 [file 12687_2026_941_MOESM2_ESM.docx]

| **Supplemental Table 1. Expanded participant demographics (n=31)** | |
| --- | --- |
| **Characteristics** | **M ± SD or n (%)** |
| **Nativity** |  |
| Yes | 26 (83.9%) |
| No | 5 (16.1%) |
| **Gender Identity^*^** |  |
| Agender | 2 (6.5%) |
| Bigender | 0 (0.0%) |
| Genderfluid | 4 (12.9%) |
| Gender nonconforming | 4 (12.9%) |
| Genderqueer | 3 (9.7%) |
| Man | 3 (9.7%) |
| Nonbinary | 15 (48.4%) |
| Transgender man | 6 (19.4%) |
| Transgender woman | 13 (41.9%) |
| Transmasculine | 7 (22.6%) |
| Transfeminine | 4 (12.9%) |
| Woman | 4 (12.9%) |
| Another Gender | 2 (6.5%; Demi-boy, Transgender) |
| **Disability description^*+^** |  |
| Attention deficit | 7 (22.6%) |
| Autism | 6 (19.4%) |
| Blind or visually impaired | 0 (0.0%) |
| Deaf or hard of hearing | 0 (0.0%) |
| Health-related disability | 4 (12.9%) |
| Learning disability | 1 (3.2%) |
| Mental health condition | 4 (12.9%) |
| Mobility-related disability | 2 (6.5%) |
| Speech-related disability | 0 (0.0%) |
| Other | 5 (16.1%) |
| **Sexual Orientation^*^** |  |
| Asexual | 0 (0.0%) |
| Bisexual | 6 (19.4%) |
| Demisexual | 4 (12.9%) |
| Gay | 2 (6.5%) |
| Lesbian | 7 (22.6%) |
| Pansexual | 6 (19.4%) |
| Straight/heterosexual | 4 (12.9%) |
| Two-spirit | 1 (3.2%) |
| Queer | 19 (61.3%) |
| Questioning/unsure | 0 (0.0%) |
| Another sexual identity/orientation | 4 (12.9%; Allosexual, Aromantic, Sapphic, Omnisexual) |
| **Employment** |  |
| Yes | 23 (74.2%) |
| No | 8 (25.8%) |
| **Insurance^*^** |  |
| Insurance through your current or former employer or union | 17 (54.8%) |
| Insurance through someone else’s current or former employer or union | 5 (16.1%) |
| Insurance purchased through insurance marketplace | 1 (3.2%) |
| Insurance purchased directly from insurance company | 1 (3.2%) |
| Medicare | 1 (3.2%) |
| Medicaid | 3 (9.7%) |
| TRICARE/military healthcare | 0 (0.0%) |
| Veterans Affairs | 0 (0.0%) |
| Indian Health Service | 0 (0.0%) |
| Don’t know | 2 (6.5%) |
| Other | 3 (9.7%) |
| **Religion importance** |  |
| Not at all | 15 (48.4%) |
| Not too important | 3 (9.7%) |
| Somewhat important | 8 (25.8%) |
| Very important | 5 (16.1%) |
| **Genomics work** |  |
| Yes | 4 (12.9%) |
| No | 27 (87.1%) |
| **Previous genomics research participation** |  |
| Yes | 4 (12.9%) |
| No | 27 (87.1%) |
| **Previous genetic testing** |  |
| Yes | 10 (32.3%) |
| No | 21 (67.7%) |
| **Household size** | 1.8 (1.1) |
| ^*^Individuals could select more than one response  ^+^Participants were only shown question if they indicated they had a disability. Denominator for percentage is total study population. | |
